# Sexually dimorphic characteristics of the small intestine and colon of prepubescent C57BL/6 mice

**DOI:** 10.1186/s13293-014-0011-9

**Published:** 2014-08-29

**Authors:** Wilma T Steegenga, Mona Mischke, Carolien Lute, Mark V Boekschoten, Maurien GM Pruis, Agnes Lendvai, Henkjan J Verkade, Jos Boekhorst, Harro M Timmerman, Torsten Plösch, Michael Müller

**Affiliations:** 1 Nutrition, Metabolism and Genomics Group, Division of Human Nutrition, Wageningen University, Wageningen, The Netherlands; 2 Center for Liver, Digestive and Metabolic Diseases, Department of Pediatrics, University Medical Center Groningen, University of Groningen, Groningen, The Netherlands; 3 NIZO food Research BV, Ede, The Netherlands; 4 Department of Obstetrics and Gynaecology, University Medical Center Groningen, University of Groningen, Groningen, The Netherlands; 5 Norwich Medical School, University of East Anglia, Norwich, UK

**Keywords:** Small intestine, Colon, Sexually dimorphism, Gene expression, Microbiota, Histone modification, DNA methylation, Epigenetics, Chromosomes, Prepubescent

## Abstract

**Background:**

There is increasing appreciation for sexually dimorphic effects, but the molecular mechanisms underlying these effects are only partially understood. In the present study, we explored transcriptomics and epigenetic differences in the small intestine and colon of prepubescent male and female mice. In addition, the microbiota composition of the colonic luminal content has been examined.

**Methods:**

At postnatal day 14, male and female C57BL/6 mice were sacrificed and the small intestine, colon and content of luminal colon were isolated. Gene expression of both segments of the intestine was analysed by microarray analysis. DNA methylation of the promoter regions of selected sexually dimorphic genes was examined by pyrosequencing. Composition of the microbiota was explored by deep sequencing.

**Results:**

Sexually dimorphic genes were observed in both segments of the intestine of 2-week-old mouse pups, with a stronger effect in the small intestine. Amongst the total of 349 genes displaying a sexually dimorphic effect in the small intestine and/or colon, several candidates exhibited a previously established function in the intestine (i.e. *Nts*, *Nucb2*, *Alox5ap* and *Retnlγ*). In addition, differential expression of genes linked to intestinal bowel disease (i.e. *Ccr3*, *Ccl11* and *Tnfr*) and colorectal cancer development (i.e. *Wt1* and *Mmp25*) was observed between males and females. Amongst the genes displaying significant sexually dimorphic expression, nine genes were histone-modifying enzymes, suggesting that epigenetic mechanisms might be a potential underlying regulatory mechanism. However, our results reveal no significant changes in DNA methylation of analysed CpGs within the selected differentially expressed genes. With respect to the bacterial community composition in the colon, a dominant effect of litter origin was found but no significant sex effect was detected. However, a sex effect on the dominance of specific taxa was observed.

**Conclusions:**

This study reveals molecular dissimilarities between males and females in the small intestine and colon of prepubescent mice, which might underlie differences in physiological functioning and in disease predisposition in the two sexes.

## Background

There is growing recognition that males and females differ with respect to basic physiology, body composition and susceptibility to and progression of a broad variety of non-communicable diseases, as well as in the response to pharmacological treatment. The molecular mechanisms underlying these sexually dimorphic effects are currently largely unresolved. Increased knowledge on these mechanisms might contribute significantly to disease prevention and treatment, for instance by optimizing dietary recommendations and pharmacological protocols in a sex-specific way.

Differences between males and females not only become clearly apparent post-puberty in response to increasing circulating hormone levels but are also present at a much younger age. During the initial phase of embryonic development, differences between males and females are already detectable [1]-[4]. Immediately thereafter, male expression of the Y chromosome *Sry* gene initiates molecular and cellular cascades in the undifferentiated gonads of males leading to differentiation of testes and divergent conversion between male and female development [5]. Insight into how the sex chromosomes contribute to the differences between males and females has strongly increased in the last decade. The male Y chromosome is relatively small (60 Mb) and contains only a limited number of genes (~60 in humans and 12 in mice) [6],[7]. Even though these genes are exclusively expressed in males, a female variant for some of them has been identified [8]. The X chromosome, which is present as one copy in males and as two copies in females, is much larger in size (155 Mb) and contains a much larger number of genes (~1,500). To ensure dosage equivalence between males and females, one of the two X chromosomes is transcriptionally silenced in females [9],[10]. X inactivation silences randomly either the paternal or the maternal X chromosome, and *Xist*, a 17-kb spliced and polyadenylated RNA with no coding capacity, plays a crucial role in this process [11]-[14]. Some genes located on the X chromosome escape silencing in female somatic cells, including a number of genes containing a homologue on the Y chromosome such as the well-known escapee *Kdm5c*[15],[16].

Genome-wide gene expression analysis has revealed that sex differences occur in a wide variety of organs and tissues in humans as well as in rodents, including the liver, heart, kidney, brain, muscle, placenta, gonads and adipose tissue [17]-[25]. Sexually dimorphic genes have been best characterized in the liver [19],[22]-[24],[26]-[32]. Most studies analysing sexually dimorphic effects have been carried out in adult tissues where sex hormones likely contribute strongly to the observed effects. Conforto and Waxman have recently shown that—although by far the most pronounced difference in gene expression was found in the livers of adult mice—a significant sexually dimorphic expression was already detectable in the prepubescent mice [26]. Similar results were reported by Kwekel and colleagues for the kidney in rats [21].

Until now, sexually dimorphic effects in the intestine have only marginally been described. Oestrogen-mediated health effects in the intestine have been reported [33]-[35], and sexually dimorphic gene expression in the small intestine (SI) has been shown in adult mice [36]. Information regarding sexually dimorphic expression in the SI at a prepubescent stage is still lacking, and no information is currently available regarding sex-regulated differences in gene expression in the colon. While in the SI food is degraded by enzymatic digestion prior to absorption, the dominant process in the colon is fermentation by microorganisms. Inoculation and colonization of the newborn sterile colon starts immediately during and after delivery [37]. The existence of a strong host-microbiota interaction is currently commonly acknowledged. Furthermore, the importance of the gut microbiota for the development of the metabolic and immune system early in life is nowadays commonly appreciated [37]. Since gene expression of the colon is directly linked to the microbiota composition of the colon lumen [38]-[40], it can be speculated that sexually dimorphic expression of genes in the colon affects the microbiota composition.

Sex-related differences in gene transcription might be attributable to various mechanisms. It could relate to the location of genes on the sex chromosomes, as well as to epigenetic marks associated with the genes. Sex-specific DNA hypersensitive sites [41] and differences in DNA methylation [23] and in histone marks [22],[42] have been reported between males and females. Whether these mechanisms function equally in different organs and are globally regulated or mediated at a gene-specific level needs to be further investigated.

Tissue-specific sexually dimorphic gene expression is not well-characterized in either the SI or the colon, particularly in prepubescent subjects. To address this gap, we performed differential gene expression profiling in the SI and colon of 2-week-old male and female mouse pups by applying microarray (MA) analysis. Pyrosequencing was performed for the genes exhibiting the strongest differential gene expression to measure whether DNA methylation differences are present in the promoter regions of the sexually dimorphic genes, thereby possibly affecting the expression state. The microbiota composition of the colon luminal content was explored by deep sequencing and investigated for possible sex differences.

## Methods

### Ethics statement

The national and institutional guidelines for the care and use of animals were followed, and the experimental procedures were reviewed and approved by the Ethics Committees for Animal Experiments of the University of Groningen, The Netherlands (Ethics registration code: 5709).

### Animals and diets

Female C57BL/6 mice (5 weeks of age) were purchased from Harlan (Horst, The Netherlands) and housed individually in the light- and temperature-controlled facility of the University Medical Center Groningen (lights on 7:00 am–7:00 pm, 21°C). The mice had free access to drinking water and were fed a semi-synthetic low-fat control diet (3.85 kcal/g; 10 E% fat, 20 E% protein, 70 E% carbohydrate; D12450B, Research Diets, New Brunswick, NJ, USA). After 6 weeks (pre-treatment period), the female mice were mated with males and, in case conceiving failed, allowed to re-mate. Mice were allowed to deliver spontaneously and were left undisturbed with their litters for 24 h. Litter sizes were standardized to five to seven pups, to ensure no litter was nutritionally biased due to lower or higher litter size. By natural circumstances, the litter size of some dams was reduced further, but not changing the overall male/female ratio significantly. Throughout pregnancy and lactation, the dams received the same diet. After 2 weeks of lactation, the offspring were sacrificed by heart puncture under isoflurane anaesthesia. The SI and colon were isolated from each mouse, snap-frozen in liquid nitrogen and stored at −80°C until further use. In total, nine female and six male mice were included derived from three different litters. Physiological and molecular effects observed in the liver of these mice have been reported previously [43].

### RNA isolation

Total RNA was isolated from colon and SI samples as described previously [43]. In brief, TRIzol reagent (Invitrogen, Breda, The Netherlands) was used and the samples were treated with DNAse and purified on columns (RNeasy Micro Kit, Qiagen, Venlo, The Netherlands), all according to the manufacturer’s instructions. Purified RNA was immediately stored at −80°C until further use. RNA concentrations were determined using the NanoDrop ND-1000 UV-vis spectrophotometer (Isogen, Maarsen, The Netherlands). RNA integrity was verified on an Agilent 2100 Bioanalyzer with the 6000 Nano Kit using the Eukaryote Total RNA Nano Assay according to the manufacturer’s instructions (Agilent Technologies, Amsterdam, The Netherlands). Samples were considered suitable for hybridization when they showed intact bands of 18S and 28S ribosomal RNA subunits, displayed no chromosomal peaks or RNA degradation products and had a RNA integrity number (RIN) above 8.0.

### Microarray hybridization and analysis

Per offspring, colon and SI samples were analysed as described previously [43], and in total, six male and six female samples were used in this analysis (three of the nine females were not included in the microarray analysis due to budget limitations). In brief, 100 ng of purified RNA was used for the preparation of labelled cDNA, applying the Ambion Whole Transcript (WT) Expression Kit (Life Technologies, Carlsbad, CA, USA) in combination with the Affymetrix GeneChip WT Terminal Labeling Kit (Affymetrix, Santa Clara, CA, USA). All samples were hybridized at one time point to Affymetrix GeneChip Mouse Gene 1.1 ST arrays according to standard Affymetrix protocols. Quality control and normalization were performed using Bioconductor software packages integrated in an on-line pipeline [44]. Normalized expression estimates of probe sets were computed by the robust multiarray (RMA) analysis algorithm available in the Bioconductor library AffyPLM using default settings [45]. Probe sets were redefined according to Dai et al. [46] and assigned to unique gene identifiers (IDs) of the Entrez Gene database, resulting in 21,187 assigned Entrez IDs. Array data were submitted to the Gene Expression Omnibus and are available under accession number GSE57516.

### Bioinformatic analysis

Of the 21,187 defined genes covered by the MA, only genes with an intensity value of ≥20 on at least five arrays, represented by at least seven probes per gene on the array and an interquartile range (IQR) ≥0.1 were selected for further analysis whereby the colon and SI were analysed separately. Pancreas-specific genes (http://biogps.org) were removed from the analysis in order to omit an effect of potential pancreatic contamination. For the SI in total 12,297 and for the colon 14,400 genes were included in the subsequent analysis; 10,958 genes met the above-described criteria in both the SI and colon. The top 1,000 most variable genes were used for principal component analysis (PCA) using MultiExperiment Viewer version 4.8.4 [47],[48] with an eigenvalue of 1.0 as a cutoff for identification of contributing components. Signal 2log ratios, which represent fold changes (FC), and related significances of change were calculated from the mean signal intensities of the female and male groups for analysis of the sex-specific differential gene expression using intensity-based moderated *t* statistics (IBMT) implementing empirical Bayes correction [49]. Resulting 2log ratios and *p* values were applied for further descriptive bioinformatic analysis of the data. Ingenuity pathway analysis (IPA; Ingenuity® Systems, www.ingenuity.com) was used to relate the MA data to networks of diseases and biological functions. Comparison of male and female expression patterns in the SI and colon on genes located on the autosomes was carried out by generating heat maps using MultiExperiment Viewer version 4.8.1 [47],[48].

### DNA isolation from the SI and colon

Genomic DNA was isolated from the SI and colon by using the DNeasy® Blood and Tissue Kit (Qiagen, Venlo, The Netherlands) as described before [50] according to the manufacturer’s instructions. The DNA was treated with RNase and eluted in Qiagen elution buffer AE. DNA purity and quantity were checked spectrophotometrically (ND-1000, NanoDrop Technologies, Wilmington, DE, USA).

### Bisulfite conversion and DNA methylation analysis

For each sample, 500 ng of genomic DNA was bisulfite-treated using the EZ-96 DNA Methylation-Gold™ Kit (Zymo Research, Irvine, CA, USA) and eluted in 14 μl of M-Elution Buffer. DNA methylation analysis was performed using PyroMark™ pyrosequencing technology (Biotage AB, Uppsala, Sweden). Primers were designed using PyroMark software, and the sequences of the primers used are listed in Additional file 1. The PCR reactions were performed in a total volume between 25 and 50 μl, and the volume of bisulfite-treated genomic DNA used was always 1/20 of the total PCR volume. PyroMark PCR Master Mix and CoralLoad Concentrate were used according to the manufacturer’s instructions, and 0.2 μM of each primer (Qiagen, Venlo, The Netherlands) was used. The following thermal cycling conditions were applied: 15 min at 95°C, followed by 45 cycles of 94°C for 30 s, tempX (gene-specific, see Additional file 1) for 30 s, and 72°C for 40 s, followed by a final elongation step at 72°C for 10 min. The PCR product was bound to Streptavidin Sepharose HP beads (GE Healthcare, Uppsala, Sweden) and purified and made single-stranded using the Pyrosequencing Vacuum Prep Tool according to the manufacturer’s instructions (Qiagen, Venlo, The Netherlands). Sequencing primers (for sequences, see Additional file 1) were annealed to the purified single-stranded PCR product, and pyrosequencing was performed using the Q24 Pyrosequencing System (Qiagen, Venlo, The Netherlands). CpG methylation was analysed with the provided software.

### Bacterial DNA extraction

DNA from all six male and nine female sacrificed mice was extracted from the freeze-dried luminal content of the colon using the method described by Salonen et al. [51]. In short, approximately 0.1 g was used for mechanical and chemical lysis using 0.5 ml buffer (500 mM NaCl, 50 mM Tris-HCl (pH 8), 50 mM EDTA, 4% SDS) and 0.25 g of 0.1-mm zirconia beads and 3-mm glass beads. Nucleic acids were precipitated by addition of 130 μl of 10 M ammonium acetate, using one volume of isopropanol. Subsequently, DNA pellets were washed with 70% ethanol. Further purification of DNA was performed using the QIAamp DNA Stool Mini Kit (Qiagen, Hilden, Germany). Finally, DNA was dissolved in 200 μl Tris/EDTA buffer, and its purity and quantity were checked spectrophotometrically (ND-1000, NanoDrop Technologies, Wilmington, DE, USA).

### Library preparation for 16S rRNA pyrosequencing

Universal primers were applied (forward primer, 5′-*CCATCTCATCCCTGCGTGTCTCCGACTCAGNNNNNN***ACTCCTACGGGAGGCAGCAG**-3′; reverse primer, 5′-*CCTATCCCCTGTGTGCCTTGGCAGTCTCAG***CRRCACGAGCTGACGAC**-3′) for amplification of the V3-V6 region of the 16S rRNA gene as described before [52]. The forward primer includes a sample-specific six-base barcode (*NNNNNN*) to tag each PCR product. The amplification PCR consists of 2 μl microbial genomic DNA, 16 μl master mix (1 μl KOD Hot Start DNA Polymerase (1 U/μl; Novagen, Madison, WI, USA), 5 μl KOD buffer (10×), 3 μl MgSO_4_ (25 mM), 5 μl dNTP mix (2 mM each), 1 μl (10 μM) of each forward and reverse primer) and 32 μl sterile water (total volume 50 μl). PCR conditions were as follows: 95°C for 2 min followed by 35 cycles of 95°C for 20 s, 55°C for 10 s and 70°C for 15 s, ending with a last step of 72°C for 10 min to ensure complete amplification of the target region. From each sample, 5 μl was electrophoresed on a 1% agarose gel and the approximately 750-bp PCR amplicon was subsequently purified using the MSB Spin PCRapace kit (Invitek, Westburg, The Netherlands) followed by a second purification step using PureLink columns (Invitrogen, Breda, The Netherlands). The concentration was checked with a NanoDrop 2000 spectrophotometer (Thermo Scientific, Wilmington, DE, USA). A composite sample for pyrosequencing was prepared by pooling 200 ng of these purified PCR products of each sample. The pooled samples were submitted for pyrosequencing of the V3-V4 region of the 16S rRNA gene on the 454 Life Sciences GS FLX platform using Titanium sequencing chemistry at GATC Biotech, Konstanz, Germany.

### 16S rRNA gene sequence analysis

Pyrosequencing data were analysed with a workflow based on QIIME v1.2, as described before [52]. Diversity metrics were calculated as implemented in QIIME v1.2. Hierarchical clustering of samples was performed using UPGMA with weighted UniFrac as a distance measure as implemented in QIIME v1.2. The Ribosomal Database Project classifier version 2.2 was performed for taxonomic classification [53]. The significance of the difference in relative abundance of specific taxa between males and females was calculated using the Mann-Whitney *U* test as implemented in SciPy [54]. Additional data handling was done using in-house developed Python and Perl scripts.

### Statistical analysis

Statistical analysis for the MA and microbiota data are described in the respective sections. Changes in DNA methylation were evaluated with an unpaired *t* test. For all tests, *p* values <0.01 were considered statistically significant.

## Results

### Gene expression in the small intestine and colon of prepubescent mice is sexually dimorphic

**Figure 1:**
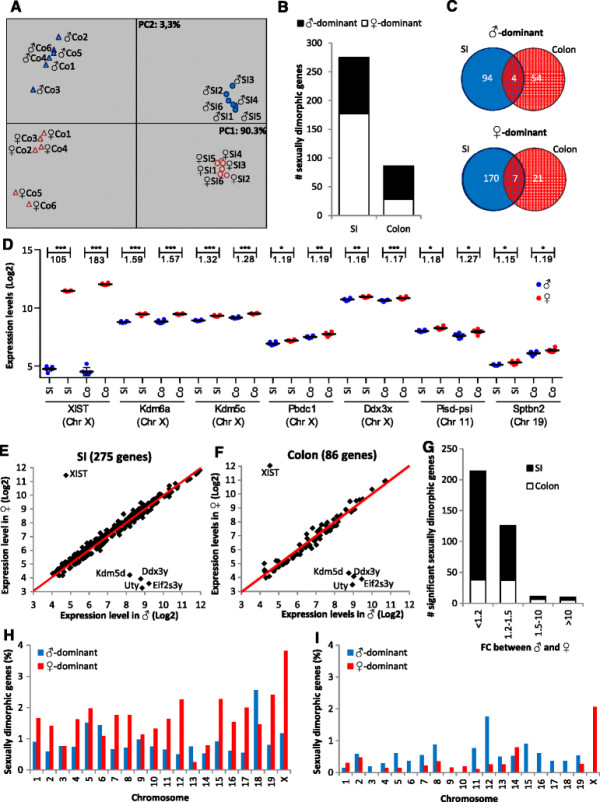
**Sexually dimorphic gene expression in the SI and colon of 2-week-old C57BL/6 mice. (A)** PCA of the top 1,000 most variable genes present in the SI or the colon separates the SI from the colon by PC1 and males from females by PC2. **(B)** In the SI and the colon, 275 and 86 genes displayed significant (*p* < 0.01) sexually dimorphic expression, respectively. **(C)** Four of the significant sexually dimorphic genes revealed male-dominant expression in both the SI and colon while seven genes showed female-dominant expression in both intestinal segments. **(D)** Expression levels of the seven female-dominant genes in the SI and colon in all individual male and female mice. Correlation between male and female expression levels of the **(E)** 275 sexually dimorphic genes in the SI and **(F)** 86 sexually dimorphic genes in the colon. **(G)** The FC difference between males and females is very low for the majority of the sexually dimorphic genes. Chromosomal localization of the sexually dimorphic genes in the **(H)** SI and **(I)** colon relative to the total number of genes localized on each chromosome in the total selection of 10,958 included in the analysis. *p < 0.01, **p < 0.001, ***p < 0.0001.

To determine differences in gene expression in the intestine between male and female mice, whole-genome expression profiling was performed on the small intestine (SI) and colon of 2-week-old male and female C57BL/6 mice using microarray analysis (MA). Principal component analysis (PCA) was applied on the top 1,000 most variable genes present in the SI or the colon. The results obtained show distinct separation of the samples in two clusters by principal component (PC) 1, accounting for 90.6% of the expression variation. As seen in Figure 1A, one cluster contains the SI and the other cluster the colon samples. In addition, the samples within each cluster are divided by PC2 accounting for 3.3% of the variation, entirely separating the males and females in both intestinal segments.

Next, genes showing significant (*p* < 0.01) sexually dimorphic gene expression were analysed in more detail for each of the two segments of the intestine separately. Of the total number of 10,958 genes remaining after filtering, 275 genes displayed significant differential expression in the SI including 98 male-dominant and 177 female-dominant genes (see Figure 1B and Additional file 2). In the colon, a smaller number of genes (86) revealed sexually dimorphic expression patterns of which 58 genes demonstrated male-dominant and 28 genes showed female-dominant expression (see Figure 1B and Additional file 3). Figure 1C shows that in total, seven genes displayed female-dominant expression in the SI as well as in the colon. Five of them are located on the X chromosome and two are autosomal genes. Of these seven genes, only *Xist* displays strong differential expression between the two sexes while for *Kdm6a*, *Kdm5c*, *Pbdc1*, *Ddx3*, *Pisd-psi* and *Sptbn2* more subtle but significant differences were observed (Figure 1D). The four genes displaying male-dominant expression in both segments of the intestine are *Kdm5d*, *Ddx3y*, *Uty* and *Eif2s3y*. All of them are located on the Y chromosome and reveal, as expected, strong differential expression since these genes are not expressed by females. Furthermore, we identified two genes showing significant sexually dimorphic expression in the two segments of the intestine but with an opposite sex effect. *Cabp2* displays female-dominant expression in the SI and male-dominant expression in the colon while for *Gm6581* male-dominant expression was found in the SI and female-dominant expression in the colon. In total, 349 different genes revealed sexually dimorphic expression in either the SI or the colon.

By analysing the sexually dimorphic effects in the SI and colon in more detail, we found that the majority of the changes are relatively subtle in both segments of the intestine (see Figure 1E, F). Evaluation of the fold changes (FC) of all sexually dimorphic genes in either the SI or the colon revealed that, in line with previously reported data in the liver, heart, adipose tissue and brain [19], the majority of the genes showed only small differences (FC <1.2) in gene expression between males and females (Figure 1G). The number of genes that differed more than 1.5-fold between males and females is very limited.

To analyse the chromosomal distribution of the sexually dimorphic genes, we calculated the chromosomal localization of the sexually dimorphic genes relative to the chromosomal localization of all 10,958 genes included in the analysis. As shown in Figure 1H, I, different distributions were found for the SI and colon, but in both segments, the strongest effects were observed on the sex chromosomes (see Additional file 4).

In summary, significant changes in gene expression were found in the SI and colon of prepubescent mice, with the highest FC between the sexes observed for genes located on the sex chromosomes.

### Inter-individual variation detected in many genes displaying sexually dimorphic expression

Next, we concentrated our analysis on the genes displaying the most pronounced sexually dimorphic effect in the SI and the colon. The top 25 genes displaying the strongest differential expression are presented in Table 1. The results reveal that, apart from the above-indicated four Y-chromosomal and X-chromosomal *Xist* genes showing strong sex-specific expression in both the SI and the colon, 20 genes are present, exhibiting a FC between 1.40 and 1.75. All these genes are located on autosomal chromosomes except for the X-chromosomal gene *Kdm6a*. Interestingly, for a number of these genes (indicated with §), intestinal expression and/or functioning has been described [55]-[59] but until now sexually dimorphic expression has not been reported.

**Table 1:** Top 25 genes displaying the strongest differential expression between males and females in the intestine

**Symbol**	**SI**	**Colon**	**Chr**	**Promoter length**	**Number of CpGs**	**Description**
Eif2s3y	−49.68^♂^	−46.96^♂^	Y	682	21	Eukaryotic translation initiation factor 2, subU 3, str. gene Y-linked
Uty	−48.21^♂^	−44.48^♂^	Y	893	28	Ubiquitously transcribed tetratricopeptide repeat gene, Y chr
Ddx3y	−28.71^♂^	−30.81^♂^	Y	643	23	DEAD (Asp-Glu-Ala-Asp) box polypeptide 3, Y-linked
Kdm5d	−15.67^♂^	−21.58^♂^	Y	757	30	Lysine (K)-specific demethylase 5D
Upk1b	−1.12^	−1.60^♂^	16	603	11	Uroplakin 1B
Wt1	−1.02^	−1.59^♂^	2	1282	104	Wilms tumour 1 homologue
Syndig1	−1.52^♂^	−1.05^	2	603 + 1,225	3 + 91	Synapse differentiation inducing 1
Nos2	1.09^	−1.41^♂^	11	673	8	Nitric oxide synthase 2, inducible
Rab42	−1.42^♂^	−1.17^	4	783	35	RAB42, member RAS oncogene family
Nts^§^	−1.41^♂^	−1.15^	10	794	6	Neurotensin
Cyp2c66	1.45^♀^	−1.07^	19	613	1	Cytochrome P450, family 2, subfamily c, polypeptide 66
Mmp25	1.43^♀^	1.05^	17	893	36	Matrix metallopeptidase 25
Slc7a11^§^	1.40^♀^	1.05^	3	829	18	Solute carrier family 7, member 11
Nucb2^§^	1.46^♀^	1.07^	7	666	36	Nucleobindin 2
Alox5ap^§^	1.44^♀^	1.13^	5	705	4	Arachidonate 5-lipoxygenase-activating protein
Gm12696	−1.13^	1.41^♀^	4			Predicted gene 12696
Cabp2	1.50^♀^	−1.06^	19	603 + 820	3 + 8	Calcium-binding protein 2
Fkbp11	1.56^♀^	1.09^	15	1041	27	FK506-binding protein 11
Mcpt1	−1.02^	1.54^♀^	14	606	5	Mast cell protease 1
Mcpt2	−1.12^	1.66^♀^	14	601	1	Mast cell protease 2
Dleu2	−1.11^	1.71^♀^	14	798 + 601	84 + 45	Deleted in lymphocytic leukaemia, 2
Ccr3	1.59^♀^	1.50^	9	730	3	Chemokine (C-C motif) receptor 3
Retnl*γ*^§^	1.72^♀^	1.52^	16	638	4	Resistin-like gamma
Kdm6a	1.59^♀^	1.57^♀^	X	693 + 601	51 + 69	Lysine (K)-specific demethylase 6A
Xist	104.49^♀^	182.91^♀^	X	1,065	29	Inactive X-specific transcripts

^♂^Significant ♂-dominant expression FC >1.4.

^♀^Significant ♀-dominant expression FC >1.4.

^FC without a significant sexually dimorphic effect.

^§^Previous studies revealed an intestine-specific function and/or expression of this gene.

**Figure 2:**
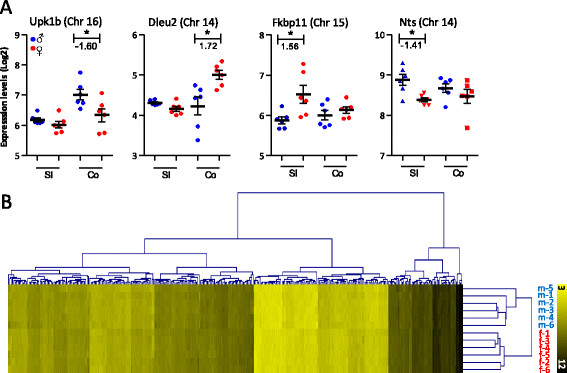
**Inter-individual variation in gene expression within the groups of male and female mice. (A)** Expression levels of *Upkb1*, *Dleu2*, *Fkbp11* and *Nts* in the SI and colon of the individual male and female mice. **(B)** Hierarchical clustering of the expression levels of the subset of 329 sexually dimorphic genes revealed separation of the males and females in two distinct clusters. *p < 0.01.

By evaluating the expression levels of the top 25 strongest sexually dimorphic genes in the individual mice in more detail, substantial variation in expression between the individual pups within the sex groups was observed. As seen in Figure 2A, mean expression values of *Upk1b*, *Dleu2*, *Fkbp11* and *Nts* significantly differed between male and female mice, but at an individual level, some of the female mice show the same expression level as a number of the male mice. The results for these genes are in contrast with the genes presented in Figure 1D, where variation between the individual mice of one sex was extremely low. Hierarchical cluster analysis (HCA) of expression levels of the subset of 329 sexually dimorphic genes revealed separate profiles for male and female mice even in the absence of the genes located on the sex chromosomes. This result indicates that, despite the inter-individual variation in expression of the sexually dimorphic genes, male and female mouse pups reveal a clearly distinct expression profile (Figure 2B).

### Differential gene expression might have functional consequences for normal functioning and disease development between males and females

Ingenuity pathway analysis (IPA) was applied to identify the biological functionality in physiology and disease as well as the functional networks of the sexually dimorphic genes. By analysing the physiological (Bio) functions in-depth, IPA revealed that the sexually dimorphic genes represented various general cellular functions in both the SI and the colon (Table 2). Regarding disease development, IPA revealed that in the SI, genes displaying sexually dimorphic expression were mainly involved in infection and inflammation, whereas in the colon also genes involved in cancer development were found to be differentially regulated (Table 2).

**Table 2:** Top Bio functions and diseases linked to the sexually dimorphic genes

**Bio functions**	***p*** **value**	**#mol**	**Diseases**	***p*** **value**	**#mol**
SI					
Cellular movement	8.04E − 14 to 6.18E − 3	67	Inflammatory response	1.23E − 12 to 6.1E − 3	60
Cellular function and maintenance	5.95E − 11 to 6.1E − 3	68	Hypersensitivity response	7.04E − 8 to 9.51E − 4	24
Cellular compromise	3.84E − 9 to 2.44E − 3	23	Infectious disease	6.11E − 5 to 5.59E − 3	29
Cell signalling	2.01E − 8 to 5.71E − 3	40	Hereditary disorder	1.16E − 4 to 2.78E − 3	6
Molecular transport	2.01E − 8 to 5.71E − 3	62	Inflammatory disease	1.16E − 4 to 3.29E − 3	43
Colon					
Cellular development	1.12E − 4 to 4.19E − 2	19	Haematological disease	6.06E − 4 to 3.36E − 2	11
Cellular growth and proliferation	1.12E − 4 to 4.19E − 2	23	Inflammatory response	1.5E − 3 to 4.19E − 2	12
Cell morphology	1.4E − 4 to 4.31E − 2	17	Cancer	1.57E − 3 to 4.21E − 2	19

*#mol* number of molecules included in the indicated Bio function or disease.

In Table 3 the top networks having *p* scores of at least 25 [60] are presented. The results obtained reveal that in the SI, sexually dimorphic expression is found for different subsets of genes involved in lipid metabolism (network identifier ID-1 and ID-2), genes playing a role in developmental networks (ID-3) and genes involved in “cell-to-cell signalling and interaction, haematological system development and function and immune cell trafficking” (ID-4). In the colon, two top networks with scores of at least 25 were found, one representing genes with a function in “cardiovascular system development and function, organismal development and tissue morphology” (ID-1) and the other one containing genes with a function in “neurological disease, physiological disorders and protein synthesis” (ID-2).

**Table 3:** Top networks of sexually dimorphic genes

**ID**	**Top networks**	**mol/score**^**a**^
SI ♂ vs ♀		
1	Lipid metabolism, small-molecule biochemistry, vitamin and mineral metabolism	24/41
	ABCG1♀,ALDH1A3♀,ALS2CR12♂,CD300LD♀,CLN8♂,COX6B2♂,CTRC♀,CYP1B1♂,CYP2C19♀,CYP4B1♀,HCK♀,HDAC11♀,	
	mir-181♀,MYBL1♂,MYBL2♀,NCAM2♂,PREX1♀,S100A1♂,SELPLG♀,SH3BP1♀,SLC46A3♂,TIFA♂,TOLLIP♂,UBA3♂	
2	Lipid metabolism, small-molecule biochemistry, haematological system development and function	18/28
	ACER3♂,ASAH1♂,BTK♀,CD22♀,LAPTM5♀,NFATC1♀,P2RX7♀,PIK3AP1♀,PIK3CD♀,PLA2G7♀,PLA2G7♀,PLA2G10♀,	
	PLCG2♀,PLD2♀,PRR7♀,RASGRP4♀,TNR♂,VPS45♂	
3	Haematological system development and function, tissue development, cellular movement	17/26
	AHR♀,CCL11♀,GNPDA1♂,HCLS1♀,IL15♂,KDM5D♂,KDM6A♀,LAMP1♂,MBD1♂,MZB1♀,P2RX1♀,PLCB2♀,PYGL♀,	
	RAC2♀,SRGN♀,TRIM5♂,Uty♂	
4	Cell-to-cell signalling and interaction, haematological system development and function, immune cell trafficking	17/26
	BET1♂,BMP7♂,Ceacam1/Ceacam2♂,DLL4♀,EGF♀,ETS1♀,FXYD5♀,HMGCS1♂,mir-148♂,MMP10♀,PARVG♀,	
	RUNX3♀,SAMHD1♀,SH3KBP1♀,SH3PXD2B♂,STAP1♀,TRAM2♂	
Colon ♂ vs ♀		
1	Cardiovascular system development and function, organismal development, tissue morphology	20/44
	CABP4♂,DDX3X♀,Dleu2♀,DLK1♂,ITGA2B♂,KLK3♀,MAP3K8♂,mir-10♂,mir-181♂,MTCH2♀,NOS2♂,NPR3♂,	
	PDGMC♂,PRKCZ♂,PRX♂,STX6♂,SYT9♂,TNMRSM19♂,TNMRSM11B♂,WT1♂	
2	Neurological disease, psychological disorders, protein synthesis	16/33
	BIVm♀,CWC22♀,EIF2S3♂,FUNDC2♀,KDM6A♀,KRT75♂,MED29♂,mir-134♂,PBDC1♀,REEP1♂,RRP12♂,SPTBN2♀,	
	STAG3♂,SURF6♀,Uty♂,WDR44♀	

Networks with *p* scores ≥25 are presented.

*♂* genes displaying ♂-dominant expression, *♀* genes displaying ♀-dominant expression.

^a^Focus molecules/score.

### Epigenetic mechanisms might contribute to differential expression of sexually dimorphic genes

**Figure 3:**
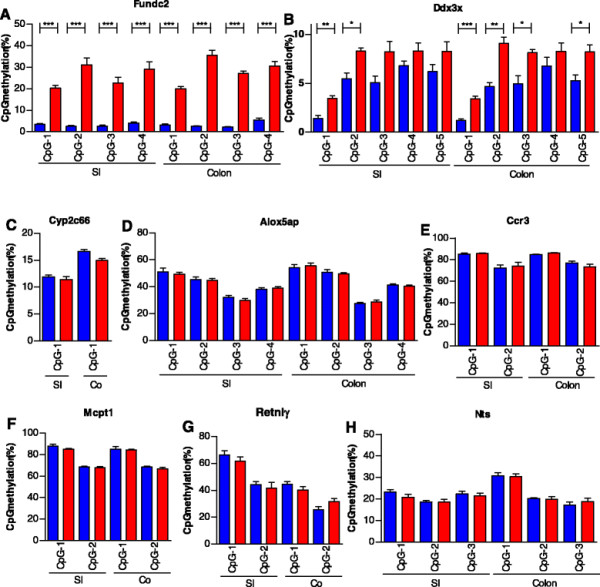
**DNA methylation analysis of the promoter regions of genes displaying sexually dimorphic expression.** Significant differential methylation was found in the promoter region of two X-chromosomal genes: **(A)***Funcd2* and **(B)***Ddx3x*. No differential CpG methylation was found in the promoter region of the autosomal genes **(C)***Cyp2c66*, **(D)***Alox5ap*, **(E)***Ccr3*, **(F)***Mcpt1*, **(G)***Retnlγ* and **(H)***Nts*, all displaying significant sexually dimorphic expression with a FC difference >1.4 between males and females. Blue bars: males; red bars: females. *p < 0.01, **p < 0.001, ***p < 0.0001.

By analysing the total subset of 349 sexually dimorphic genes in more detail, we identified 9 genes exhibiting a function in epigenetic processes (see Table 4). Interestingly, apart from *Mbd1*, which is known for its binding to methylated DNA, all genes have histone-modifying activity. It can be speculated that the sexually dimorphic expression of these histone modifiers might contribute to the differential expression of a subset of the other sexually dimorphic genes. Since the amount of intestinal tissue of 2-week-old mouse pups is very limited and all available material was used for RNA and DNA isolation, we were not able to examine histone modifications in the SI and colon tissue in more detail. Since we could not measure histone modifications, we analysed DNA methylation as a surrogate marker of epigenetic regulation. DNA methylation has been shown to be involved in silencing of genes located on one of the two X chromosomes in somatic cells [61]. We measured DNA methylation by applying pyrosequencing on the promoter regions of a number of X-chromosomal genes. The results presented in Figure 3A, B clearly show significantly enhanced methylation for all four CpGs analysed in the promoter of the *Fundc2* promoter and for part of the five CpGs analysed in the *Ddx3x* promoter of female mouse pups. We used the top 25 of sexually dimorphic genes to evaluate the presence of promoter methylation as a potential regulator of differential expression between the sexes. The promoter regions of the genes were identified by applying Genomatix analysis, and the CpG content of each promoter was determined. As shown in Table 1, strong differences in the number of CpGs present in the different promoters were found, varying from a few CpG dinucleotides (i.e. *Cyp2c66*, *Alox5ap*, *Cabp2*, *Mcpt1*, *Mcpt2*) to very CpG dense promoter regions (i.e. *WT1*, *Syndig1*, *Dleu2*, *Kdm6a*). Pyrosequencing analysis revealed no differential methylation between males and females in any of the CpG sites studied that are present in the promoter region of *Cyp2C66*, *Alox5ap*, *Ccr3*, *Mcpt1*, *Retnlγ* and *Nts*, representing a subset of genes containing a relative low CpG content (see Figure 3C–H and Additional file 5).

**Table 4:** Genes displaying sexually dimorphic expression and exhibiting a role in epigenetic processes

**Symbol**	**FC-SI**	**FC-Colon**	**Description**	**Chr**	**Epigenetic activity**
Mbd1	−1.13^♂^	−1.03	Methyl-CpG-binding domain protein 1	18	Binding to methylated DNA
Phf20	1.02	−1.14^♂^	PHD finger protein 20	2	Histone acetylation
Uty	−48.21^♂^	−44.48^♂^	Ubiqu. transcribed tetratricopeptide repeat gene, Y chr	Y	Histone demethylation
Kdm5d	−15.67^♂^	−21.58^♂^	Lysine (K)-specific demethylase 5D	Y	Histone demethylation
Kdm6a	1.59^♀^	1.57^♀^	Lysine (K)-specific demethylase 6A	X	Histone demethylation
Kdm5c	1.32^♀^	1.28^♀^	Lysine (K)-specific demethylase 5C	X	Histone demethylation
Bcorl1	1.15^♀^	1.04	BCL6 co-repressor-like 1	X	Associates with HDAC activity
Hdac11	1.14^♀^	−1.11	Histone deacetylase 11	6	Histone deacetylation
Piwil4	1.17^♀^	−1.03	Piwi-like RNA-mediated gene silencing 4	9	Histone methylation

^♂^Significant ♂-dominant expression; ^♀^significant ♀-dominant expression.

In summary, our data suggest potential involvement of mediators of histone modification in the regulation of sexually dimorphic effects, but we did not detect significant changes in CpG methylation in the promoter regions of a small subset of the top 25 sexually dimorphic genes between the two sexes.

### No sex effect on bacterial community compositions of the colon

**Figure 4:**
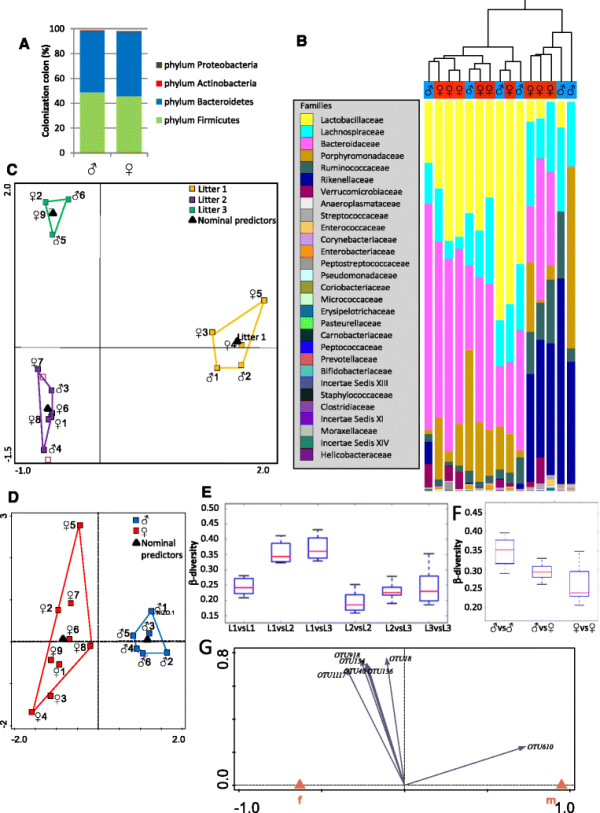
**Microbiota composition analysis of the colonic luminal content revealed no significant differences between male and female prepubescent mice. (A)** Bacteriodetes and Firmicutes dominate the colon lumen in 2-week-old male and female pups. **(B)** Hierarchical clustering using weighted UniFrac as a distance measure of the male and female mouse pups revealed no clustering of the sexes. **(C)** RDA plots show microbiota differences based on litter origin, but **(D)** only marginal and not significant differences were found between male and female mice. Boxplot diagrams of the beta-diversity showed **(E)** a more distinct difference of the mice of litter 1 compared to litters 2 and 3 but **(F)** no significant differences between males and females. **(G)** OTU610 (classified as *Syntrophococcus*) was identified to being most uniquely associated and hence predictive for the male sex.

To evaluate the intestinal microbiota composition of the 2-week-old pups, deep sequencing of the 16S rRNA gene was applied on colonic luminal content collected from the mice. As seen in Figure 4A, the colon lumen of both male and female mice is predominantly colonized by Bacteroidetes and Firmicutes and, in addition, very small fractions of Actinobacteria and Proteobacteria were measured. Evaluating the families colonizing the colon of the 2-week-old suckling mice revealed the presence of a variety of families in the colon lumen. The results presented in Figure 4B showed that hierarchical clustering using weighted UniFrac as a distance measure separates the samples of all 15 mice included in the analysis into two major clusters, but it did not separate the males from the females. Redundancy analysis (RDA) revealed that the three litter origins in which the mice were nursed during the 2 weeks of their life have a dominant and highly significant effect on microbiota composition. This effect is visualized in the RDA plot presented in Figure 4C showing a clear separation in microbiota composition of the mice of the three litters whereby gender was included as a covariant. Multivariate statistics taking litter origin into account as a covariant showed a trend for sex effect on microbiota composition (Figure 4D), but this effect was not significant (*p* > 0.01). Beta-diversity metrics calculated from the three different litters (Figure 4E) further confirmed the observation that litter but not sex (Figure 4F) has a strong effect on bacterial community compositions. Interestingly, for males, one specific operational taxonomic unit (OTU610 classified as *Syntrophococcus*) was identified as the most unique taxon associated with males (Figure 4G), which was confirmed by random forest analysis identifying it as the strongest predictor for males (see Additional file 6). Follow-up studies containing a larger sample size and isolated at different time points are required to validate and further explore this result.

Taken together, our data reveal that litter origin has the strongest impact on bacterial community composition and no significant effect of gender was observed. However, we cannot exclude that sex has an effect on the dominance of specific taxa.

## Discussion

### Sex-specific functionality and susceptibility to disease development of the intestine

By applying MA analysis, we identified sexually dimorphic genes in both segments of the intestine of prepubescent C57BL/6 mice, with the most abundant effect observed in the SI. The vast majority of genes displayed only small expression differences between males and females. This effect is in line with previously reported sexually dimorphic effects found in adult liver, brain, muscle and adipose tissue of 334 mice [19]. A small subset of genes displayed more pronounced sexually dimorphic effects including *Nts*, *Nucb2*, *Slc7a11*, *Alox5ap* and *Retnlγ*, which are genes that have previously been linked to intestinal expression and/or functioning. Our results reveal male-dominant expression of *Nts*, a gene expressed by the enteroendocrine N cells in the intestine. The 13-amino acid gene product is excreted and serves multiple functions including stimulating pancreas and biliary secretion [62],[63] and affecting gastric, SI and colonic motility [59],[64]. *Nucb2*, which displays female-dominant expression in the SI, is expressed at different positions of the gastrointestinal system including the duodenum [65]. Nefstatin-1 is the 82-amino acid excreted peptide derived from *Nucb2* and has been shown to play a role in food intake regulation by inducing an anorexic signal in the hypothalamus [66]. *Slc7a11* mediates the transfer of amino acids over the cellular membrane, and its expression in the intestine has been described before [56]. Our study revealed female-dominant expression of *Slc7a11* in the SI of the prepubescent mice. *Alox5ap* is involved in the synthesis of leukotrienes which are lipid-signalling molecules derived from arachidonic acid and showed higher expression in the SI of female mice. Leukotrienes are known to initiate and amplify inflammation, and expression of *Alox5ap* in intestinal cells has previously been reported [57]. Finally, *Retnlγ* has been reported to be expressed in the intestine and has been suggested to play a role in intestinal tract-mediated regulation of insulin sensitivity [67] and in inflammatory processes [68]. *Retnlγ* showed significant female-dominant expression in the SI of the 2-week-old mice. In addition, the list of genes revealing more subtle changes in gene expression between males and females included additional transporters and genes involved in lipid metabolism. In conclusion, our results indicate that sexually dimorphic expression of genes might induce distinct functioning of both segments of the intestine in males and females.

The results obtained by IPA carried out on the significantly differentially expressed genes imply that also disease development might be regulated differently between the two sexes. Predominantly, genes involved in infectious disease and inflammatory processes were differentially expressed between the two sexes and—again—this effect was most pronounced in the SI. In addition, the top 25 list of genes exhibiting the most pronounced sexually dimorphic expression in either the SI or colon included four genes (*Nos2*, *Mcpt1*, *Mcpt2*, *Ccr3*) that carry out functions in immune response and inflammation. In humans, one of the most frequently occurring diseases of the intestine is inflammatory bowel disease (IBD), which includes two chronic diseases that cause intestinal inflammation: Crohn’s disease and ulcerative colitis (UC). Both are multifactorial disorders and factors playing a causal role in disease development are genetic predisposition, the intestinal microbiota, the immune system and environmental factors—in particular nutrition [69]-[71]. There is currently no consensus regarding the effect of sex on disease development of both Crohn’s disease and UC. While some of the epidemiological studies did observe sexually dimorphic effects, other studies did not [72]. Interestingly however, several of the identified sexually dimorphic genes containing a function in inflammatory processes have previously been linked to IBD including *Ccr3*[73], *Ccl11*[74], *Ncf1*[75] and *Tnfr*[76]. Apart from genes playing a role in immune response and inflammation, we identified cancer-relevant genes in the colon showing sex-differential expression using IPA. Furthermore, the top 25 of most differentially expressed genes includes two genes that have previously been linked to colorectal cancer: *Mmp25*[77] and *WT1*[78]. Taken together, our data reveal that genes involved in disease development of the intestine in adults show differential expression between the sexes. It should be taken into account that in this study, we analysed prepubescent mice. Samples collected at other ages, as well as human samples, are required to further explore the molecular mechanisms that might be responsible for differences in intestinal disease development between males and females.

### Inter-individual variation within the sexes

Even though the results of the MA analysis revealed significant sex differences for the expression of numerous genes, most fold changes are relatively low. It might be anticipated that, when a larger sample number would have been analysed, the number of genes revealing significant sexually dimorphic expression might have been higher. Contributing to the relatively low fold changes, we found substantial inter-individual variation between the individual mice within one sex, especially for a subset of genes displaying stronger expression differences between males and females (FC 1.4–1.75). Although C57BL/6 is an inbred mouse strain, strong phenotypic variations between male mice at different ages in response to exposure to a high-fat [79] or medium-fat (Rusli et al.: A weekly alternating diet between caloric restriction and medium-fat protects the liver from development of NAFLD in middle-aged C57BL/6J mice, submitted) diet have been described previously by us. Nonetheless, hierarchical clustering of the significantly differentially expressed genes—after exclusion of genes located on the sex chromosomes—still separated the male and female mice in two distinct groups. This result indicates that male and female expression profiles of the sexually dimorphic genes are distinct. This effect is independent of the variation in expression within the two sexes and is detectable even in the absence of sex-linked genes. Studies including larger numbers of mice are required to evaluate inter-individual variation of the sexually dimorphic genes in more detail.

### Sexually dimorphic effects pre- and post-puberty

In our study, we analysed sexually dimorphic effects in the intestine of prepubescent mice and found that in these 2-week-old mice pups, already substantial changes in gene expression were detectable in particular in the SI. We have previously reported physiological and molecular differences in the liver between the two sexes of these young mice in response to maternal exposure to a Western-style diet [43]. By comparing the significantly sexually dimorphic genes between the intestine and the liver, we found that most of the genes displaying differential expression between the sexes in the two segments of the intestine were also found in the liver. However, overall the overlap in genes displaying significant sexually dimorphic expression between the liver and the intestine was very low (Additional file 7). By comparing differential expression of male and female adult mice in different tissues, Yang and colleagues observed high tissue specificity for the sexually dimorphic genes in the liver, brain, adipose tissue and muscle [19]. By analysing the common functional categories of the sexually dimorphic genes, they found that only steroid and lipid metabolism were shared between the different tissues. Interestingly, we also found differential expression of genes contributing to lipid networks in the SI. The reason why we did not observe an effect in steroid metabolism might be due to the fact that this study has been carried out in 2-week-old pups when hormonal differences between the sexes are still extremely low. Evaluation of the 349 sexually dimorphic genes revealed that this list did not include well-established oestrogen- or androgen-responsive genes.

Our analysis included samples collected of prepubescent mice. In previous studies of Conforto and Waxman [26] and Kwekel et al. [21], sexually dimorphic gene expression was analysed in pre- as well as post-pubertal animals. Both studies revealed that, although sexually dimorphic effects are detectable in pre-pubertal animals, the effects are much more pronounced in the adult phase of life. These results imply that also the difference between males and females for the two segments of the intestine might be much stronger in adult mice. On the other hand, Kwekel and coworkers noticed that part of the sexually dimorphic effect detected in the youngest animals disappeared at older ages [21], indicating differences in the kinetics of the developmental processes. Interestingly, according to data published by Mank and colleagues, the kinetics over the life cycle of sexually dimorphic genes in chicken differ between males and females [24]. Whether similar effects occur in the intestine needs to be explored in further studies.

### Variation in microbiota composition between the mice is driven by litter origin but not by sex

The newborn gut is essentially sterile. Inoculation and colonization of the colon starts already during delivery, and time is required to allow the first settlers to establish a dense microbial population. The final population colonizing the colon includes thousands of bacterial species that belong to a limited number of phyla. In the adult intestinal lumen, Bacteroidetes and Firmicutes are the dominant phyla, but during the colonization phase, bifidobacteria species (members of the Actinobacteria phylum) are usually dominant in breastfed infants [37]. However, in our 2-week-old mouse pups, an extremely limited amount of bifidobacteria was observed and the colon microbiota was almost exclusively made out of Bacteroidetes and Firmicutes. Our results indicated that variation between bacterial communities was dominated by litter origin while no significant difference between males and females was found. Most likely, the microbiome of their mothers is a critical component of the offsprings’ microbiome at this early pre-weaning phase and might dominate over other potential modifiers. However, we cannot exclude that sex has an effect on the dominance of specific taxa. These observations are consistent with previous reports, which showed that gut microbiota differences become evident in adult male and female mice and are driven by androgen hormones, but are absent in prepubescent mice [80],[81]. Furthermore, various other influencing factors including cage and maternal effects have been reported previously [82],[83].

### Localization on the sex chromosomes together with epigenetic effects might be responsible for the sexually dimorphic gene expression

As indicated above, sexually dimorphic gene expression is much more robust in the adult phase of life than during the prepubescent phase due to circulating sex hormones. Although very low levels of sex hormones are already present in early life, there is increasing understanding that also hormone-independent pathways of sexual differentiation exist [84]. Alternative mechanisms responsible for molecular regulation of sexually dimorphic effects are (1) expression of genes located on the sex chromosomes and (2) epigenetic differences between the sexes. Our study revealed relative abundance of sex chromosomal localization amongst the sexually dimorphic genes. Of the total subset of 349 intestinal sexually dimorphic genes, 19 were located on the X chromosome and 4 on the Y chromosome. Furthermore, our results showed that, consistent with previously reported data [85], the majority of the X-chromosomal genes displayed female-dominant expression and apart from the well-known escapees *Xist*, *Kdm6a* and *Kdm5c*, only marginal expression differences between males and females were detected. The Y-linked copy of *Kdm6a* and *Kdm5c*, *Uty* and *Kdm5d*, respectively [8], revealed strong expression in the intestine of the 2-week-old mice pups. Many of the sexually dimorphic genes located on the sex chromosomes display a regulating function in transcription (i.e. *Btk*, *Ddx3x*, *BcorL1*, *Uty*) or translation (*Eif2s3y*), and it can be speculated that these genes contribute to the differential expression of the autosomal genes.

Other potential mechanisms responsible for sexually dimorphic expression are epigenetic modifications. Previous studies have provided evidence that changes in histone modifications [22],[42] as well as alterations in DNA methylation [23] might contribute to differential gene expression between the two sexes. Our study displayed differential expression of a subset of genes involved in regulating histone modifications supporting the previously reported results. By analysing the promoter regions of the top 25 strongest sexually dimorphic genes, we observed high diversity in the CpG content ranging from extremely high CpG dense to just one or a few CpGs. We analysed DNA methylation of various genes containing low or medium CpG dense promoter regions and found no significant changes in DNA methylation in any of the 14 analysed CpG sites between males and females. Weber et al. identified a link of CpG density of promoter regions with their methylation status [86] which might be responsible for our findings instead of sex-differential effects. Moreover, pyrosequencing has the limitation to analyse only selected CpGs/regions and is not a genome-wide approach. Therefore, differential DNA methylation might occur on CpGs in the promoter regions that have not been included in our analysis. Furthermore, changes in gene expression might also be regulated by changing the methylation status of CpGs present in enhancer regions or in the gene body of the genes, which were not included in our analysis. Genome-wide DNA methylation analysis to investigate CpG methylation on a more extensive scale is required to determine the involvement of DNA methylation as underlying regulatory mechanisms of sexually dimorphic gene expression in the SI and colon.

## Conclusions

Our study revealed sexually dimorphic genes in both segments of the intestine. Functional analyses of these genes pointed towards differences in normal physiological functioning as well as disease development between males and females. No significant differences in bacterial community composition were found, although sex-specific taxa might exist. Since prepubescent animals have been used in this study, we can conclude that, even in the absence of circulating sex hormones and in the presence of identical microbiota in the colon lumen, there is intrinsic sex-specific gene regulation. Since the intestine fulfils a central role in whole-body health, the observed molecular sexually dimorphic effects might contribute to the differences in basic physiology, body composition and susceptibility to and progression of a broad variety of non-communicable diseases between males and females.

## Abbreviations

FC: fold change

HCA: hierarchical clustering analysis

IBD: inflammatory bowel disease

IBMT: intensity-based moderated *t* statistics

IDs: identifiers

IPA: ingenuity pathway analysis

IQR: interquartile range

MA: microarray analysis

PC: principal component

PCA: principal component analysis

RDA: redundancy analysis

RMA: robust multiarray

SI: small intestine

UC: ulcerative colitis

## Competing interests

Jos Boekhorst and Harro Timmerman are affiliated with NIZO food research BV. The other authors declare that they have no competing interests.

## Authors' contributions

HJV, MiM, TP and WTS conceived and designed the experiments. MGMP, MoM, AL and CL performed the experiments. WTS, MVB, JB and HMT analysed the data. WTS wrote the paper. MVB assessed the quality control of microarrays. MoM, HJV, MiM, TP, MVB, JB and HMT provided valuable feedback on the manuscript. All authors read and approved the final manuscript.

## Additional files

## Supplementary Material

Additional file 1: **Primer sequences used for DNA methylation analysis by pyrosequencing.** Primer sequences that have been used to measure DNA methylation by pyrosequencing in the promoter region of *Nts*, *Cyp2c66*, *Alox5ap*, *Mcpt1*, *Ccr3*, *Retnlg*, *DDX3X* and *Fundc2*. Click here for file

Additional file 2: **Genes displaying significant (*p* < 0.01) sexually dimorphic expression in the SI.** Overview of the expression levels in each individual mice in the SI and colon, FC and Limma *p* values of the 275 genes displaying significant differential expression between males and females in the SI. Click here for file

Additional file 3: **Genes displaying significant (*p* < 0.01) sexually dimorphic expression in the colon.** Overview of the expression levels for each individual mice in the SI and colon, FC and Limma *p* values of the 86 genes displaying significant differential expression between males and females in the colon. Click here for file

Additional file 4: **Chromosomal localization of sexually dimorphic genes in the SI and colon.** Overview of the absolute and relative frequency of sexually dimorphic genes on the chromosomes. Click here for file

Additional file 5: **Position of the analysed and not analysed CpGs in the promoter regions of the genes selected for pyrosequencing analysis.** For none of the analysed positions, a significant difference in methylation between males and females was detected (*p* in all cases >0.01). Click here for file

Additional file 6: **Male-dominant OTUs detected in the colonic luminal content of 2-week-old C57BI/6 pups.** OTU_610 was identified as a predictor for male mouse pups. Click here for file

Additional file 7: **Genes displaying significant (*p* < 0.01) sexually dimorphic expression in the SI, colon and/or liver of 2-week-old C57BL/6 mice.** Overview of the 507 genes displaying significant differential expression between males and females in the SI, colon and/or liver of 2-week-old mice and their chromosomal localization. Click here for file
