# Supplementary material for: Sexually dimorphic characteristics of the small intestine and colon of prepubescent C57BL/6 mice
Source: Biol Sex Differ. 2014 Aug 29;5:11. doi: 10.1186/s13293-014-0011-9 (PMC4169057; doi:10.1186/s13293-014-0011-9)
Supplement: Additional file 5: — Position of the analysed and not analysed CpGs in the promoter regions of the genes selected for pyrosequencing analysis. For none of the analysed positions, a significant difference in methylation between males and females was detected (p in all cases >0.01). [file s13293-014-0011-9-S5.pptx]

## Slide 1
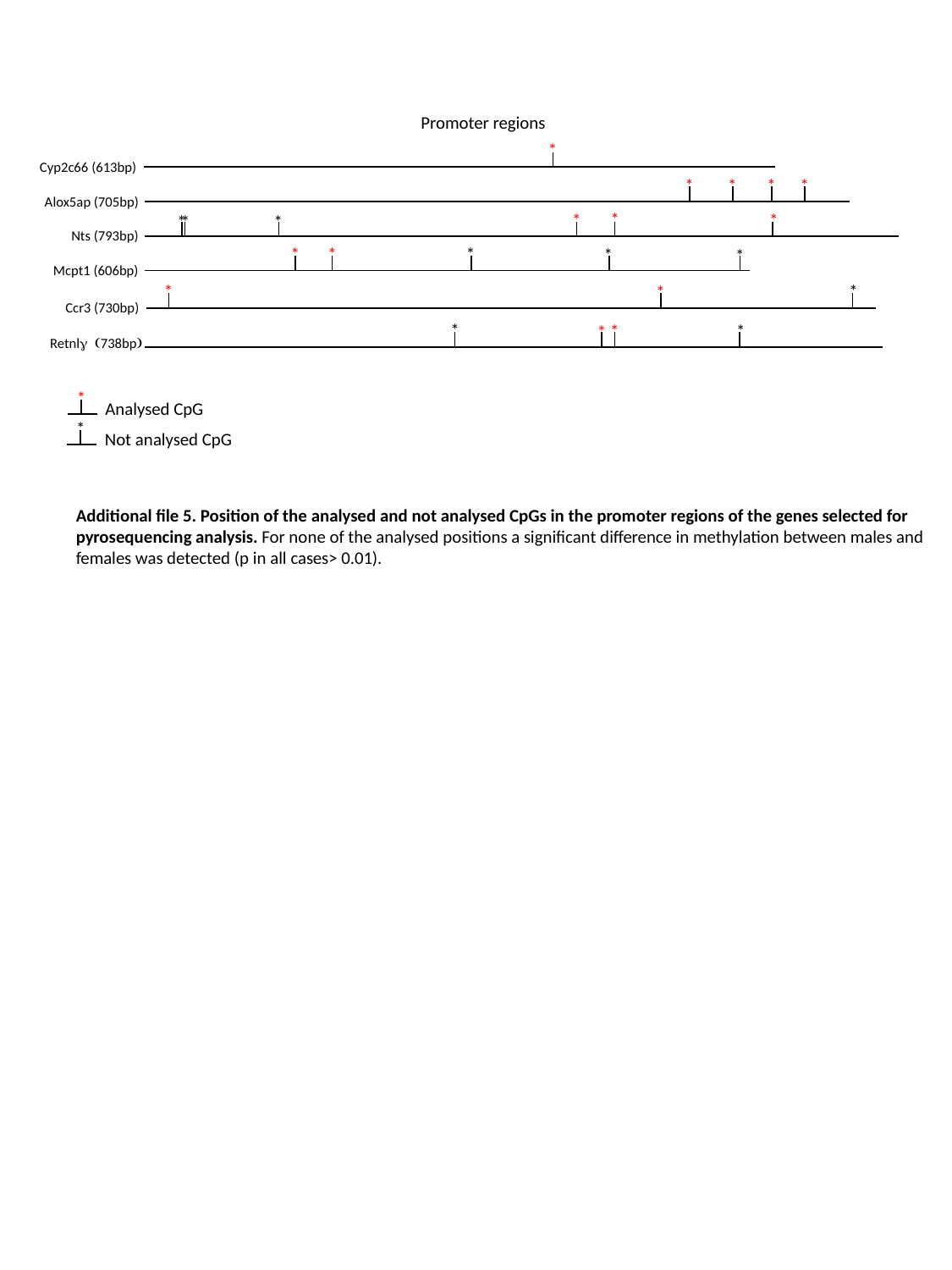

Promoter regions
*
Cyp2c66 (613bp)
*
*
*
*
Alox5ap (705bp)
*
*
*
*
*
*
Nts (793bp)
*
*
*
*
*
Mcpt1 (606bp)
*
*
*
Ccr3 (730bp)
*
*
*
*
Retnlg (738bp)
*
Analysed CpG
*
Not analysed CpG
Additional file 5. Position of the analysed and not analysed CpGs in the promoter regions of the genes selected for pyrosequencing analysis. For none of the analysed positions a significant difference in methylation between males and females was detected (p in all cases> 0.01).
