# Supplementary material for: Sexually dimorphic characteristics of the small intestine and colon of prepubescent C57BL/6 mice
Source: Biol Sex Differ. 2014 Aug 29;5:11. doi: 10.1186/s13293-014-0011-9 (PMC4169057; doi:10.1186/s13293-014-0011-9)
Supplement: Additional file 6: — Male-dominant OTUs detected in the colonic luminal content of 2-week-old C57BI/6 pups. OTU_610 was identified as a predictor for male mouse pups. [file s13293-014-0011-9-S6.pptx]

## Slide 1
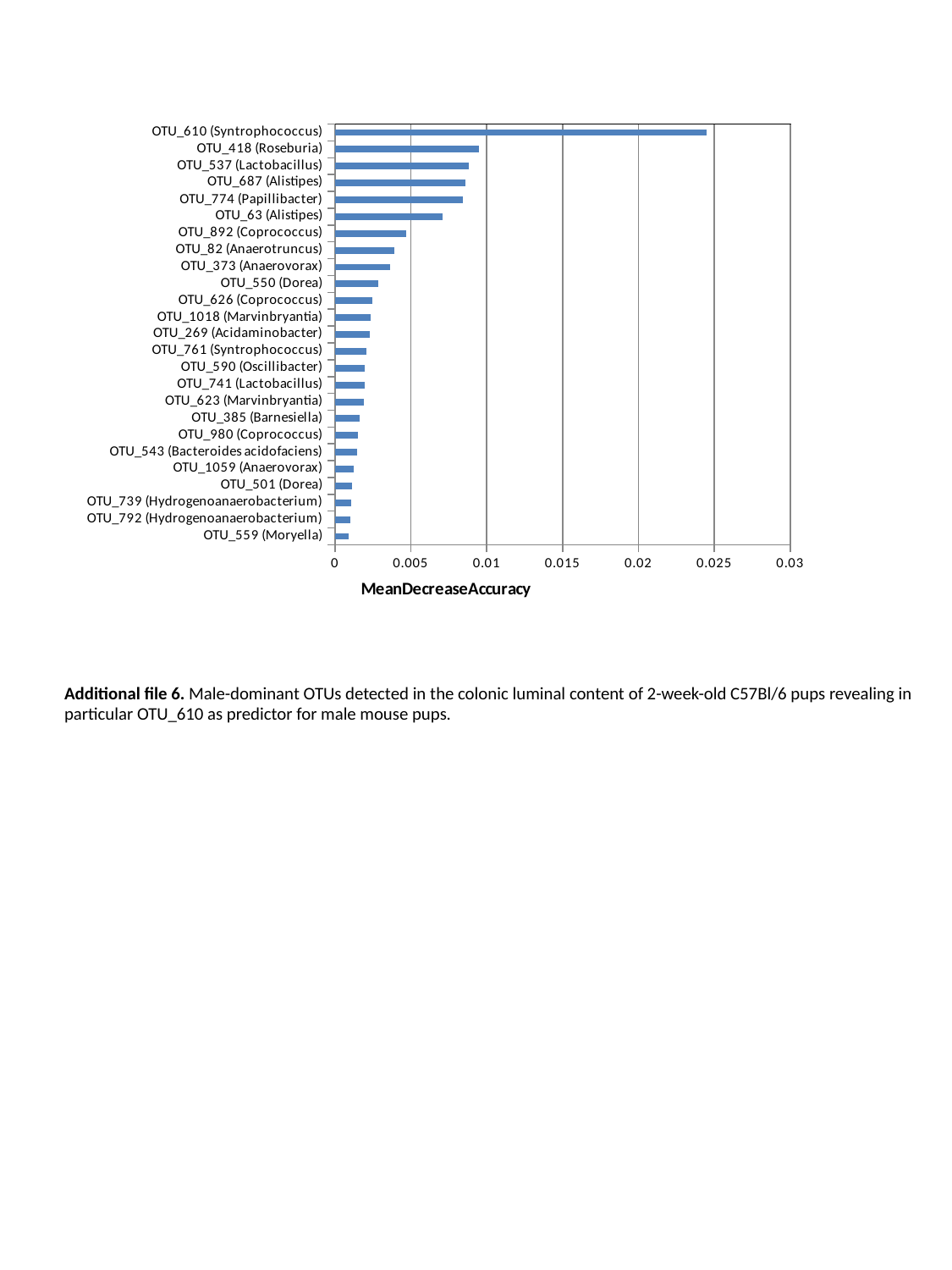

### Chart
| Category | MeanDecreaseAccuracy |
|---|---|
| OTU_559 (Moryella) | 0.0009 |
| OTU_792 (Hydrogenoanaerobacterium) | 0.000993 |
| OTU_739 (Hydrogenoanaerobacterium) | 0.001086 |
| OTU_501 (Dorea) | 0.001148 |
| OTU_1059 (Anaerovorax) | 0.00121 |
| OTU_543 (Bacteroides acidofaciens) | 0.001481 |
| OTU_980 (Coprococcus) | 0.00151 |
| OTU_385 (Barnesiella) | 0.001618 |
| OTU_623 (Marvinbryantia) | 0.001886 |
| OTU_741 (Lactobacillus) | 0.001936 |
| OTU_590 (Oscillibacter) | 0.001979 |
| OTU_761 (Syntrophococcus) | 0.002076 |
| OTU_269 (Acidaminobacter) | 0.002279 |
| OTU_1018 (Marvinbryantia) | 0.002326 |
| OTU_626 (Coprococcus) | 0.00245 |
| OTU_550 (Dorea) | 0.00286 |
| OTU_373 (Anaerovorax) | 0.003644 |
| OTU_82 (Anaerotruncus) | 0.003931 |
| OTU_892 (Coprococcus) | 0.004664 |
| OTU_63 (Alistipes) | 0.007115 |
| OTU_774 (Papillibacter) | 0.008439 |
| OTU_687 (Alistipes) | 0.008579 |
| OTU_537 (Lactobacillus) | 0.008848 |
| OTU_418 (Roseburia) | 0.009479 |
| OTU_610 (Syntrophococcus) | 0.024511 |Additional file 6. Male-dominant OTUs detected in the colonic luminal content of 2-week-old C57Bl/6 pups revealing in particular OTU_610 as predictor for male mouse pups.
